# Growth patterns, metabolic indicators and osteoarticular status in the Lusitano horse: A longitudinal study

**DOI:** 10.1371/journal.pone.0219900

**Published:** 2019-07-17

**Authors:** Maria J. Fradinho, Luísa Mateus, Nuno Bernardes, Rui J. B. Bessa, Rui M. Caldeira, Graça Ferreira-Dias

**Affiliations:** 1 Centre for Interdisciplinary Research in Animal Health, Faculty of Veterinary Medicine, University of Lisbon, Lisboa, Portugal; 2 Faculty of Veterinary Medicine, University of Lisbon, Lisboa, Portugal; Massey University, NEW ZEALAND

## Abstract

Development of a healthy musculoskeletal system is of high concern for horse breeders and users. A longitudinal field study was performed in order to: (i) evaluate growth patterns and long-term changes on bone quality, bone metabolism, growth factors and metabolic variables in the Lusitano horse; and (ii) retrospectively assess whether these changes were related with radiographic findings regarding osteochondrosis-like lesions (OC) at the onset of training. Thirty-four Lusitano foals born and raised at four stud-farms, were periodically weighed (BW), and measured (withers height-WH) from birth to 36 months of age. On the same days, blood samples were collected for determination of osteocalcin, bone alkaline phosphatase, insulin-like growth factor I (IGF-I), leptin, insulin, glucose, parathyroid hormone (PTH), calcium, phosphorus and magnesium plasma concentrations, and quantitative ultrasound measurements were performed on the right third metacarpal bone (McIII). At the end of the study horses underwent radiographic examination of the four fetlocks, hocks and stifles. According to their radiographic status (OC negative *vs*. OC positive), Richards growth function was adjusted to BW and WH data. Instantaneous BW and WH growth rates (BW IADG and WH IADG) were calculated for each foal, from the resolution of the first derivative of growth models for seven age-classes. The presence of radiographic findings compatible with OC at the onset of training was associated with changes in BW and WH growth rates. Positive horses presented higher BW IADG from six to 18 months of age and lower WH IADG before 45 days of age (P<0.001). Speed of sound measurements (SOS), bone markers, growth factors and other metabolic variables change markedly with age (P<0.01). OC positive horses tended to have lower SOS values at the lateral region of McIII, lower IGF-I, and higher insulin and PTH concentrations (P<0.1). This study provides indirect evidence that monitoring foals’ growth during the first year of life may be of assistance in managing the occurrence of OC. Further studies with a higher number of animals and a controlled feed intake should be pursued.

## Introduction

In the equine athlete, development of a healthy locomotor system is fundamental for the success and longevity of sports performance. Thus, one of the main concerns of the horse breeding industry is the knowledge of the most adequate growth pattern for each breed and purpose, as well as the factors that influence skeleton development.

Growth and development in the sport horse is particularly high during the first year of life [1]. Depending on the breed, 58% to 67% of adult body weight and 87 to 90% of adult withers height are reached at 12 months of age, pointing out to an early development of bone tissue [2–4]. However, body weight gain and skeletal growth are closely related with feed intake level [5, 6].

Rapid growth in the first stage of a horse’s life was identified as one of the factors that is associated with the occurrence of skeletal development disorders [7, 8]. Among these disorders, those affecting articular cartilage, in particular osteochondrosis (OC), have been recognized as a major cause of economic losses for the horse industry [9]. Osteochondrosis is a dynamic disturbance in the natural process of endochondral ossification during growth, with a complex multifactorial etiology [10, 11]. Genetic predisposition and environmental factors such as exercise/biomechanical stress, nutrition, growth rate and local/endocrine disturbances in the cartilage have been generally implicated in this condition [12–15].

Over the past years, some non-invasive techniques have been developed in order to assess bone quality, bone metabolism and the adaptive response of the skeleton to development or mechanical stimuli [16]. Among them, quantitative ultrasonography (QUS) is a nonionizing radiation methodology that can provide an indication of bone quality, as it reflects mineral density and the mechanical properties of cortical bone [17–19]. Another non-invasive technique widely used to monitor changes of bone metabolism, is the assessment of biochemical markers in body fluids [16]. Osteocalcin (Oc) and bone alkaline phosphatase (BALP) are two proteins associated with osteoblastic activity, which have been widely used as bone formation markers in the horse [20, 21]. These biomarkers have been also related with the detection of bone or osteoarticular diseases [22–24]. Furthermore, the hormonal action of Oc as a mediator between bone and energy metabolism was investigated. In this process, Oc is regulated by leptin and insulin, two hormones involved in energy homeostasis [25–26].

Other hormonal factors may have interpretative value regarding bone growth and metabolism. Insulin-like growth factor I (IGF-I) is involved in the regulation of longitudinal bone growth, acting locally in the growth plate as a stimulator of chondrocyte proliferation and cartilage maturation [27–29]. In addition, parathyroid hormone (PTH) and plasma mineral concentrations have been investigated in the context of nutritional imbalances implicated in skeletal development disorders [30, 31].

Nowadays, due to their functional and behavioral characteristics, the reputation of the Lusitano as a sport and leisure horse is increasing worldwide [32]. Hence, gathering reliable information under farm conditions, about the growing process and its relation to osteoarticular quality of the foals is of major importance for breeders and users. Therefore, the aims of the present study were: (i) to evaluate growth patterns and long-term changes on bone quality, bone metabolism, growth factors and other metabolic variables in the Lusitano horse; and (ii) to retrospectively assess whether these changes were related with radiographic findings regarding OC-like lesions at the onset of training.

## Materials and methods

The present study received the approval of the Ethical Committee of the Faculty of Veterinary Medicine, University of Lisbon, Portugal. All the animals were handled with care during the experimental procedures.

### Animals and management

This investigation was part of a longitudinal study aiming to investigate growth and development of the Lusitano horse and was conducted over a 5-year period, in four stud-farms, located at the main region of Lusitano breeding, in Portugal. Thirty-four foals (12 colts and 22 fillies) born in 2006 (n = 19), in 2007 (n = 9) and in 2008 (n = 6) were monitored from birth to 36 months of age. The foals were born between February and May (Feb, n = 13; Mar, n = 9; Apr, n = 8; May, n = 4).

All the animals, mares and foals, were kept on pasture throughout the study and had *ad libitum* access to water. The stud-farms are located between latitude 38°88’ to 39°29’ N and longitude 07°67’ to 08°87’W and, according to Köppen-Geiger classification, are under the influence of a Mediterranean climate (Csa) [33]. The mean annual rainfall on stud-farm location range from 652 to 830 mm. Mean temperatures range from 8.7° in January to 24.1°C in August, with the lowest values observed in January (-4.5°C) and the highest in August (45.2°C) [34]. Floristic composition was typical of natural rain fed pastures of these regions, with a high biodiversity. Besides a mixture of native grasses and legumes, some forbs and weeds were also present. Among grasses (Poaceae), the main *genera* includes *Lolium* spp., *Phalaris* spp., *Bromus* spp., *Agrostis* spp. and *Poa* spp.. In the legume family (Fabaceae), plants from the *genera Trifolium* spp., *Vicia* spp., *Melilotus* spp., *Ornithopus* spp. and some annual species of *Medicago* were identified. Pasture samples were collected in the four stud-farms during the spring months (March, April and May) for chemical composition analysis and nutritive value evaluation (S1 Table). In this study pasture productivity was not measured, but dry matter production in similar pastures of the same regions ranged from 40 kg.ha^-1^.d^-1^ in February to 90 kg.ha^-1^.d^-1^ in April, and decrease to 50 kg.ha^-1^.d^-1^ in May [35]. The stoking rate in all the stud-farms was generally low (< 0.5 head/ha).

According to lactation stage and pasture availability, mares of two stud-farms were supplemented once a day with commercial compound feeds and with grass hay or cereal straw from foaling until weaning. On the other two stud-farms mares were only supplemented with grass hay in periods when pasture was scarce. Foals were not creep fed, although some of them had access to their dam´s concentrate. The weaning occurred on average at seven and half months of age (222±33d, mean±SD). Foals were kept in paddocks with different areas depending on the stud-farm, and were group-fed with compound feeds and grass hay. After the post-weaning, foals returned to pasture (between winter and the beginning of spring), and continued to be supplemented along the study, also with compound feeds and/or grass hay, according to pasture availability. As mare and foals were group-fed, it was not possible to measure with precision the daily amounts of ingested feeds. However, the main nutritional characteristics of compound feeds are available on S2 Table. Routine vaccination and deworming programs were practiced in the four stud-farms.

### Body measurements

Foals’ body weight (BW) and withers height (WH) were assessed monthly on their first year of life, every two months on the second year, and every three months from 24 to 36 months of age. Body weight was determined using a portable electronic scale (Iconix, FX15, New Zealand), which accuracy was regularly checked. The measurement error of the scale was 0.5 kg. In the same days, WH was measured with a standard measuring stick from the ground to the highest point of the withers. All measurements were taken at a similar time of day and by the same operator.

### Blood sampling and analysis

On the days of BW and WH assessment, between 8.00h and 11.00h and before any compound feed was given, blood samples (≈ 18 mL) were collected from 27 foals (11 colts and 16 fillies), by jugular venipuncture into heparinized tubes (Monovette Li-Heparin, SARSTEDT AG & Co., Nümbrecht, Germany) for determination of Oc, BALP, IGF-I, leptin, insulin, glucose, PTH, calcium (Ca), phosphorus (P_i_) and magnesium (Mg) plasma concentrations. All blood samples were transported to the laboratory on ice and were centrifuged at 2,000 × g, at 4°C, for 15 minutes. Plasma samples were stored at –20°C until analysis.

The concentrations of Oc and BALP were measured by using two specific competitive immunoassays (METRA Osteocalcin EIA and METRA BAP EIA, both from QUIDEL Corporation, San Diego, USA), previously used and validated for the horse [24, 36, 37]. For determination of IGF-I, a commercial kit (DSL 10–5600 Active IGF-I ELISA, Webster, Texas, USA) was used based on its validation for equine plasma [38]. This IGF-I test is an enzymatically amplified “one-step” sandwich-type immunoassay. The assay includes a previous extraction step in which IGF-I was separated from its binding protein. Plasma leptin concentrations were determined by radioimmunoassay using a Multi-Species Leptin RIA kit (Linco, Millipore Corporation, Billerica, USA), widely used and validated for the horse [39, 40]. Insulin concentrations were determined with a commercial specific immunoassay kit (Mercodia Equine Insulin, Mercodia AB, Uppsala, Sweden) validated for the measurement of equine insulin [41]. The PTH concentrations were determined with a commercial kit (Intact PTH ELISA, Immunodiagnostic Systems Ltd, Boldon, UK). This two-site immunoenzymo-metric assay that was developed for the quantification of the intact parathyroid hormone (1–84), uses an N-terminal specific mouse monoclonal anti-PTH (1–34) in the solid phase and a purified goat anti-PTH (39–84) coupled to the enzyme horseradish peroxidase. Glucose and total Ca, P_i_ and Mg concentrations were measured by colorimetric methods in an auto-analyzer Kone Optima (Therma Clinical Labosystems, Vantaa, Finland) with appropriate commercial kits (Bradford, Kemia Cientifica S.A., Madrid, Spain and Thermo Electron Oy, Clinical Chemistry and Automation Systems, Vantaa, Finland). All the assays were performed according to the manufacturer’s protocols.

### Bone assessment by quantitative ultrasonography

Quantitative ultrasound measurements were performed on the foals which blood was collected (n = 27), every two months from birth to 24 months of age, and every three months from 24 to 36 months of age. A final measurement was made on the same day of the radiographic examination. Measurements were performed with a quantitative ultrasound multisite device (Sunlight EQUS, BeamMed, Ltd., Petah Tikva, Israel) developed to measure the speed of sound (SOS) in axial transmission mode through bone [17]. Before any SOS measurements were made, the accuracy of the system was calibrated using a phantom (provided by the manufacturer), to obtain temperature corrected SOS values. Static measurements with the probe in axial position were performed on the mid-section of the dorsal and lateral regions of the right third metacarpal bone (McIII) of each animal, according to the methodology previously described [18, 19]. Silicon oil was used as a coupling medium, without shaving. Horses were squared-up, standing still on the four limbs, with no sedation. All measurements were performed by the same operator.

### Radiographic evaluation

At the end of the study (after the 36 months of age) and matching the onset of training, 31 animals (10 males and 21 females) were radiographed with a portable unit (three of the initial 34 foals were sold before the radiographic examination). Radiographic projections were made using a portable high frequency x-ray generator with 2.4 kW allowing for 100 kV and 40 mAs and a Digital Radiography (DR) Flat Panel of Amorphous Selenium receptor connected to a laptop with appropriate software. Radiographic examinations were performed to every fetlock (front and hindlimbs), hocks (tarsus) and stifles, in a total of 218 joints. This examination included 12 views: lateromedial views of the four fetlocks; lateromedial, dorsolateral-plantaromedial oblique and dorsomedial-plantarolateral oblique views of both tarsus and caudolateral-craniomedial views of both stifles. The radiographic findings were evaluated by an experienced veterinarian in order to classify the presence or the absence of OC-like lesions, based on the scoring system proposed by Dik et al. [10]. Although this scoring system was originally made for stifle and hock lesions, in the present study it was also used for fetlocks. Since the main objective was to register the presence or absence of lesions (i.e. evidence of fragments or subchondral indentations), the adaption of the scoring system proposed by Dik et al. [10] was considered appropriate, as lesion location and clinical relevance were beyond the scope of this work. An example of the characteristic lesions is provided as supporting information (S1 Fig). At the time of the radiographic examinations all the horses were observed for the possible occurrence of clinical signs.

### Statistical analysis

In order to assess growth and development of the foals, individual adjustments were made to BW and WH data using Richards function, *y* = *A*(1 –*b*.exp(-*kt*))^*M*^, where the variable *y* is described as a function of age *t*. The adjustments were made using the NLIN procedures of SAS (SAS 9.3 Institute Inc., Cary, NC, USA) with the Marquardt iterative method. The parameters of Richards’ equation can be biologically interpreted. Thus, *A* is the asymptotic value of *y* as age (*t*) approached infinity, and is commonly interpreted as the mean mature size; *b* is a scaling parameter that adjusts for situations where *y*_0_ and/or *t*_0_ do not equal to 0 (for example, when only postnatal observations are available and *t*_0_ is taken as birth); *k* is a maturing index, establishing the earliness with which *y* approaches *A* (large *k* values indicate early maturing individuals and small *k* values indicate late maturing individuals); *M* determines the point of inflection where the estimate growth rate changes from an increasing to a decreasing function (for 0 < *M* < 1, *M* is undefined) [42–45].

Based on radiographic status of the horses (negative vs. positive OC) two groups were formed and growth models for BW and WH were adjusted for each group. In order to evaluate differences between the two models, a sum of squares reduction test was performed. The instantaneous rate of gain (BW instantaneous average daily gain (BW IADG), kg/d or WH instantaneous average daily gain (WH IADG), cm/d) at time *t* (*t* = days of age) was calculated from the resolution of the first derivative of Richards equation with respect to time (*δy/δt)*: *y*’ = *MAkb*.exp^(-*kt*)^ (1 –*b*.exp−^*kt*^)^*M*^ (1 –*b*.exp−^*kt*^)^-1^. Seven age-classes were considered from birth to 36 months: <45 days, 3mo [60–120 d], 6mo [150-210d], 12mo [335-395d], 18mo [480-600d], 24mo [670-790d] and 36 mo [1035-1155d]. For each horse and for each age-class, the first derivative was calculated for the average age (in days) in which measurements were performed, because most of the age-classes encompass more than one measurement.

Body weight IADG (kg/d) and WH IADG (cm/d) from birth to 36 months of age were analyzed with a mixed model considering repeated measures on time (<45days, 3mo, 6mo, 12mo, 18mo, 24mo and 36 mo). Age-class, group and its interaction were included as fixed effects in the model and an autoregressive covariance matrix was used. The same methodology was used for the analysis of SOS and blood variables, considering the two groups of horses based on their radiographic OC status. In this last analysis, only 24 animals were considered because three horses were sold before the radiographic examination.

When significant differences were detected, the Tukey-Kramer test was used to evaluate the differences among means. Statistical significance was considered when P < 0.05. Differences among means with a P-value < 0.10 and > 0.05 were considered to be indicative of a trend for selected variables. To evaluate the relationship between BW and girth measurements (taken in a previous study performed by our team [4]) Spearman's correlation coefficients were calculated (S5 Table).

All results are presented as lsmeans±SEM, unless stated otherwise.

## Results

### Growth and development

The mean and the range of parameter estimates obtained for the individual growth models that were fitted to horses’ BW and WH are presented in Table 1. Convergence criteria were met for all the models, and the mean of the calculated coefficient of determination (R^2^) showed good fit adjustments for both, BW (0.981) and WH data (0.994). However, the range of values observed for each parameter presented a wide individual variability. In particular, the values obtained for the *k* parameter, both for BW and WH, showed a large range in the maturity index even within the four stud-farms (see S3 and S4 Tables). Body weight and girth measurements were highly correlated (*r* = 0.989; P<0.0001) (see S5 Table).

**Table 1:** Descriptive statistic of the parameter estimates of the individual growth models fitted to body weight and withers height of Lusitano horses (n = 34).

Measure ^a^	Individual parameter estimates ^b^ Mean (range)				R^2^ ^c^ Mean (range)	RSD ^d^ Mean (range)
	*A*	*b*	*k*	*M*		
BW (kg)	544.0 (427.0–721.9)	0.9635 (0.7580–0.9998)	0.00135 (0.00060–0.00266)	0.671 (0.428–1.580)	0.981 (0.951–0.997)	16.5 (7.4–30.1)
WH (cm)	160.3 (152.1–173.1)	0.9472 (0.7630–0.9990)	0.00166 (0.00041–0.00345)	0.163 (0.101–0.295)	0.994 (0.985–0.998)	1.2 (0.9–1.9)

^a^ BW–body weight; WH–withers height.

^b^ *A*–asymptotic value for BW / WH as age approaches infinity (interpreted as mean BW / WH at maturity); *b*–scaling parameter that defines the degree of maturity when age = 0 d (intercept on y axis); *k*–maturing index (rate that establishes the spread of the curve along time axis); *M*–determines the point of inflexion of the curve (for 0 < *M* < 1, M is undefined).

^c^ R^2^ correspond to a pseudo R^2^, calculated as 1 –(SS(Residual) / SS(Total _corrected_)).

^d^ RSD–residual standard deviation.

### Radiographic status and growth patterns

The radiographic status and the radiographic findings by site of the 31 horses that underwent radiographic evaluation are presented in Table 2. Thirteen (41.9%) of the examined animals presented radiographic findings compatible with OC lesions and four of them showed bilateral lesions in both limb joints. No other radiographic articular lesions were identified and none of these horses presented clinical signs, such as effusions or lameness.

**Table 2:** Radiographic status and radiographic findings by site in 31 Lusitano horses.

Number of horses			Site of lesions	N° of lesions according to grade of severity^c^				N° of horses^d^
Total	Negative^a^	Positive^b^		G1	G2	G3	G4	
31	18	13	Metacarpophalangeal joint	1	1	1	-	3
			Metatarsophalangeal joint	1	5	4	1	8
			Tibiotarsal joint	2	2	2	-	5
			Femorotibial joint	-	-	-	1	1

^a^ Negative–Without any radiographic findings compatible with OC lesions

^b^ Positive–With radiographic findings compatible with OC lesions

^c^ Grade of severity: G1 –minimal; G2 –mild; G3 –moderate; G4 –severe (adapted from [10])

^d^ Four horses presented lesions bilaterally, in both limb joints.

The growth models that were adjusted to BW and WH data according to the radiographic status of the horses (group negative, n = 18 *vs*. group positive, n = 13) are presented in Table 3. Based on the adjustment of Richards function, the models obtained for the two groups were significantly different for both measurements, showing different patterns of growth in what concerns BW (P<0.001) and WH (P<0.01). The estimated maturing index (*k* parameter value) of the BW model was clearly superior in the OC positive group of horses.

**Table 3:** Body weight and withers height growth models of Lusitano horses according to radiographic status (negative *vs*. positive OC) (n = 31).

Measure ^a^	RX OC status	Parameters ^b^				R^2^ ^d^	RSD ^e^	*P* value ^f^
		*A* (± SE^c^)	*b* (± SE^c^)	*k* (± SE^c^)	*M* (± SE^c^)			
BW (kg)	Negative	497.8 ± 16.8	0.984 ± 0.012	0.00137 ± 0.0002	0.569 ± 0.049	0.927	31.1	< 0.001
	Positive	493.5 ± 17.3	0.906 ± 0.060	0.00200 ± 0.0004	0.893 ± 0.177	0.938	30.6	
WH (cm)	Negative	157.8 ± 0.9	0.956 ± 0.014	0.00175 ± 0.0002	0.153 ± 0.011	0.966	2.7	< 0.01
	Positive	159.4 ± 1.1	0.923 ± 0.027	0.00184 ± 0.0002	0.185 ± 0.020	0.953	3.3	

^a^ BW–body weight; WH–withers height.

^b^ *A*–asymptotic value for BW/WH as age approaches infinity (interpreted as mean BW/WH at maturity); *b*–scaling parameter that defines the degree of maturity when age = 0 d (intercept on y axis); *k*–maturing index (rate that establishes the spread of the curve along time axis); *M*–determines the point of inflexion of the curve (for 0 < *M* < 1, M is undefined).

^c^ SE–approximate standard error.

^d^ R^2^ correspond to a pseudo R^2^, calculated as 1 –(SS(Residual) / SS(Total _corrected_)).

^e^ RSD–residual standard deviation.

^f^ *P* value obtained under an *F* distribution, using the result of a sum of squares reduction test in order to compare differences between the two models (Negative *vs*. Positive).

Table 4 represents BW IADG and WH IADG for seven age-classes, which were calculated from the models presented in Table 3. An interaction between age-class and group was found for both variables (P<0.0001). Positive horses presented lower BW IADG in the first age-class, but the opposite was observed from the six to 18 months, indicating a higher rate of gain during this period, when compared to the OC negative animals (P<0.001). The WH IADG of positive horses were only lower than that of negative ones in the class <45 days (P<0.001).

**Table 4:** Body weight and withers height instantaneous average daily gain of Lusitano horses (n = 31) from birth to 36 months of age according to radiographic status (negative *vs*. positive OC).

Variable	OC group status	Age-class							Significance of fixed effects		
		<45d	3mo	6mo	12mo	18mo	24mo	36mo	Age-class	Group	Age-class × Group
BW IADG (kg)	Negative	1.436±0.009 A	0.811±0.008	0.561±0.008 A	0.343±0.008 A	0.238±0.008 Y	0.172±0.008	0.092±0.008	< .0001	< .0001	< .0001
	Positive	0.948±0.010 B	0.779±0.010	0.625±0.009 B	0.411±0.009 B	0.291±0.009 Z	0.193±0.010	0.092±0.010			
WH IADG (cm)	Negative	0.343±0.003 A	0.145±0.003	0.081±0.003	0.039±0.003	0.023±0.003	0.015±0.003	0.006±0.003	< .0001	0.0064	< .0001
	Positive	0.284±0.004 B	0.149±0.003	0.088±0.003	0.043±0.003	0.027±0.003	0.017±0.003	0.008±0.003			

BW IADG–body weigh instantaneous average daily gain; WH IADG–withers height instantaneous average daily gain. Values represent lsmeans±SEM

Capital letters indicate significant differences between groups in the same age-class (Tukey-Kramer adjustment, A, B: *P*<0.0001; Y, Z: *P*<0.01).

### Changes in SOS measurements and blood variables according to radiographic status

Speed of sound measurements on the dorsal region of the McIII were only influenced by age (P<0.01), but there was a trend for a difference between groups in what concerns SOS measurements on the lateral region, with lower values for the OC positive group (positive, 4160±20 m/s *vs*. negative, 4212±20 m/s; P = 0.077) (Table 5).

**Table 5:** Speed of sound measurements and plasmatic concentrations of osteocalcin, bone alkaline phosphatase, insulin-like growth factor I, leptin, insulin and glucose of Lusitano horses (n = 24*) from birth to 36 months of age according to the radiographic status.

Variable	Age-class							Significance of fixed effects		
	<45d	3mo	6mo	12mo	18mo	24mo	36mo	Age-class	Group	Age-class × Group
SOSD (m/s)	3833±31 a	3911±24 ab	3962±25 b	3958±24 b	3914±24 ab	3895±24 ab	3921±23 ab	0.0057	0.1660	0.7178
SOSL (m/s)	4026±36 a	4160±24 b	4222±25 bc	4259±25 c	4215±23 bc	4209±24 bc	4212±24 bc	< .0001	0.0767	0.9087
Oc (ng/mL)	98.6±5.5 c	85.4±5.0 c	59.9±4.9 b	39.5±5.1 a	29.2±4.9 a	35.4±5.0 a	26.2±5.7 a	< .0001	0.4751	0.3227
BALP (U/L)	299±17.7 d	230±16.4 c	200±16.1 bc	201±15.9 bc	129±16.0 a	150±16.1 ab	127±17.5 a	< .0001	0.5267	0,8940
IGF-I (ng/mL)	406±32.8 cd	442±30.3 d	359±29.7 bcd	354±30.2 bcd	228±29.7 a	290±30.2 abc	239±33.5 ab	< .0001	0.0838	0.7447
Insulin (μIU/L)	3.11±1.78 ab	1.73±1.28 a	5.29±1.33 ab	9.71±1.43 b	1.24±1.33 a	1.70±1.33 a	1.38±1.44 a	0.0001	0.0998	0.2188
Glucose (mmol/L)	9.42±0.19 d	7.46±0.18 c	6.17±0.17 b	5.37±0.18 a	4.99±0.18 a	5.16±0.18 a	5.00±0.20 a	< .0001	0.2045	0.6730
Leptin (ng/mL)	2.35±0.19 bc	2.04±0.16 abc	1.57±0.16 a	1.82±0.15 ab	1.88±0.16 ab	2.44±0.19 bc	2.69±0.18 c	0.0002	0.2195	0.5232

* Three of the 27 animals which SOS measurements were made and blood samples collected, were sold before the RX examination; OC–osteochondrosis; SOSD–speed of sound on dorsal region of the third metacarpal bone; SOSL–speed of sound on lateral region of the third metacarpal bone; Oc–osteocalcin; BALP–bone alkaline phosphatase; IGF-I–insulin-like growth factor I; Values represent lsmeans±SEM; Lowercase letters indicate significant differences within a row (Tukey-Kramer adjustment, *P*<0.05).

Plasma concentrations of bone markers were not different between OC positive and negative horses (Table 5). A significant effect of age was observed for both, Oc and BALP (P<0.001). IGF-I plasma concentrations were influenced by age and there was also a trend for lower values in the OC positive group (positive, 299.9±24.5 ng/mL *vs*. negative, 362.1±24.1 ng/mL; P = 0.084). A sharp decrease in IGF-I concentrations was observed from 12 to 18 months of age (Table 5). In what concerns insulin, there was a significant effect of age (P<0.001). Insulin increased, from three to 12 months of age, and decreased on the following months (P<0.05). A trend for higher values of insulin concentrations was observed in the OC positive group (positive, 4.29±0.67 μIU/L vs. negative, 2.62±0.69 μIU/L; P = 0.099) (Table 5). Plasma glucose concentrations were similar between OC positive and negative horses. Glucose decreased from <45days to 12 months of age, maintaining these steady levels until the end of the study (Table 5). Leptin plasma concentrations were only influenced by age (P = 0.0002). Values decreased from <45days to 6 months of age and increased in the last two age-classes (Table 5).

On Table 6, a great variation was observed on PTH plasma concentrations. Parathyroid hormone concentrations were influenced by age (P<0.05) and a trend for higher values was observed in the OC positive group (positive, 33.7±4.1 pg/mL *vs*. 22.4±4.1 pg/mL; P = 0.063) (Table 6). Concerning Ca plasma concentrations, there was a trend for an interaction between age and OC group (P = 0.078), but differences between age-classes were only observed on the negative group (P<0.05). Phosphorus and magnesium plasma concentrations were only influenced by age (P<0.001) with higher values observed at the youngest age-classes (Table 6).

**Table 6:** Plasmatic concentrations of parathyroid hormone, calcium, phosphorus and magnesium of Lusitano horses (n = 24*) from birth to 36 months of age according to the radiographic status.

Variable	OC group status	Age-class							Significance of fixed effects		
		<45d	3mo	6mo	12mo	18mo	24mo	36mo	Age-class	Group	Age-class × Group
PTH (pg/mL)		38.9±6.1	29.6±5.5	17.4±5.4	26.9±5.6	20.7±5.3	35.2±5.5	27.6±6.0	0.0292	0.0626	0.5522
Ca (mmol/L)	Negative	2.57±0.06 a	2.70±0.05 ab	2.76±0.05 ab	2.67±0.06 ab	2.73±0.05 ab	2.90±0.05 b	2.74±0.06 ab	0.0334	0.7802	0.0783
	Positive	2.74±0.06	2.82±0.06	2.65±0.05	2.69±0.05	2.69±0.05	2.77±0.06	2.76±0.07			
P_i_ (mmol/L)		2.31±0.06 d	2.12±0.05 d	1.80±0.05 c	1.59±0.05 b	1.47±0.05 ab	1.62±0.05 bc	1.29±0.06 a	< .0001	0.2440	0.5523
Mg (mmol/L)		0.85±0.02 cd	0.86±0.02 d	0.80±0.02 abc	0.77±0.02 ab	0.82±0.02 bcd	0.76±0.02 a	0.79±0.02 abc	< .0001	0.1506	0.7936

* Three of the 27 animals which blood samples were collected, were sold before the RX examination; OC–osteochondrosis; PTH–parathyroid hormone

Ca–calcium; Pi−phosphorus; Mg–magnesium; Values represent lsmeans±SEM; Lowercase letters indicate significant differences within a row (Tukey-Kramer adjustment, *P*<0.05).

## Discussion

The mean values found for each parameter of the growth models are consistent with the parameter estimates of BW and WH growth functions previously reported for the Lusitano breed [4]. According to this study, which involved a higher number of animals (n = 121) including the present sub-sample, Lusitano horses showed a slower growth rate for BW, when compared with other sport breeds. In contrast, the WH growth rate was similar to those presented by early maturing breeds, like the Thoroughbred [3].

In the present study, radiographic examinations to assess the osteoarticular status regarding the presence or absence of radiographic findings compatible with OC-like lesions were only performed when the animals were three years old. Thus, considering the dynamics of this condition and the fact that over 18 months of age most of the OC lesions stabilize, the present results can be considered reliable in what concerns their status (positive *vs*. negative OC) [9, 10, 46]. However, taking into account the small number of animals involved in this field study, any extrapolations for the Lusitano breed should be taken with care. Furthermore, comparisons within this breed or between populations of other breeds in what concerns estimates of OC prevalence based in radiographic studies, can only be made when factors like age and the number of joints are properly standardized [9]. In fact, there is a lack of radiographic surveys on the Lusitano horse with a high number of animals, under standardized conditions. Although an in depth discussion regarding OC prevalence on this breed is beyond the scope of the present study, our results regarding the lesions found in the tibiotarsal joint are in agreement with the findings of Baccarin et al. [47], who observed a persistence of 16.2% of lesions in the same joint, in 18 months old Lusitano foals. Considering the global radiographic results in our study, a higher number of lesions in the metacarpal and metatarsal phalangeal joints were also found, confirming the observations previously reported for Lusitano stallions [48, 49].

Recently, a multifactorial approach was used in some field studies, in order to investigate the etiologic factors of equine OC [50, 51]. However, only one study has explored growth parameters as a potential risk factor [51]. In the present work different growth patterns were observed for OC positive and negative horses. Lower IADG were observed for both measures in the positive group before 45 days of age, but these differences disappear after 3 months of age, showing an early change in the growth rates. The highest BW IADG observed in the OC positive group between six and 18 months, reflects an acceleration in the growth rate during this period, which is probably related to feeding practices. In fact, variable amounts of concentrate feeds were introduced in foals’ diet during the weaning and post-weaning periods. But because foals were group fed, it was not possible to accurately measure individual intakes which is a limitation of the present study. Previously, in a controlled experiment with different nutritional levels, an association between OC and fast development of some skeletal segments was shown, being WH at an early age, the variable most frequently implicated [13]. Also a cohort study with sport breed weanlings identified WH at 30 days of age and the slope of WH in the first six months after birth, as risk factors for the presence of juvenile osteochondral conditions (which includes OC) [52]. Additionally, the same group of researchers concluded that a rapid growth in girth perimeter together with irregular exercise conditions were associated to a poor osteoarticular status in the young foal [53]. In that study, foals were not weighed, but a close and well- known relationship between girth and BW measurements in the horse has been often described, making girth as one of the linear measurements that are commonly used in weight prediction equations [45, 54]. In our study a high correlation was found between BW and girth measurements. Therefore, the higher BW maturing index observed in the OC positive group could also reflect an earlier and higher growth in girth perimeter.

The concept of bone quality as assessed by QUS generally encompasses, bone density and bone mechanical properties. To the best of our knowledge there are no reports regarding a possible relationship between OC radiographic findings and *in vivo* SOS measurements on the same animals, although the potential applications of this technique on longitudinal studies concerning growth and development of the equine bone [17, 55]. Bone mineral density of excised samples from McIII, as assessed by dual-X ray absorptiometry, was found to be lower in foals with high OC scores [56]. Furthermore, cannon bone width rate gain was positively correlated with a higher incidence of developmental orthopaedic disease (DOD) [13]. This group of researchers suggested that a faster intramembranous ossification rate (responsible for the periosteal apposition and widening of long bones), could be a predisposing factor to DOD, including OC. In fact, during the periosteal apposition of primary or new bone or in situations of high bone formation, the collagen fibrils could be laid down in a disorganized manner different from the lamellar pattern commonly found in cortical bone, assuming a different microstructure called woven bone [57, 58]. Decreased SOS values on the dorsal region of the McIII were also associated to a high periosteal fibre apposition related to an increase in bone modelling process in two-year old exercised Thoroughbreds [18]. Because mechanical properties of cortical bone are greatly influenced by mineral density and by microstructural organisation of the solid matrix [57], the trend for lower SOS values observed at the lateral region of McIII, may reflect some changes in the general quality of cortical bone in OC positive animals.

In the present study, plasma concentrations of Oc and BALP did not differ between groups regarding OC radiographic status, which is in agreement with the results obtained in healthy and OC positive Hanoverian foals [59]. Nevertheless, our results are not consistent with those reported in other studies, where some positive correlations were found between Oc concentrations and the occurrence of OC [22, 23]. However, the use of different methodologies and data analysis in those studies should be taken into account. For example, the study performed by Billinghurst et al. [22] did not included unaffected foals. Therefore, the analysis of serum bone markers was achieved in reference to the severity of OC in euthanized foals, which included three score methods (macroscopic OC severity score, total OC lesion count, and total OC radiographic scores in the hock plus stifle joints). Also the positive correlations between Oc levels at two weeks of age and the occurrence of OC found in the study of Donabédian et al. [23] included the analysis of the total number of lesions detected radiographically and the necropsy score in a subsample of foals. In our study, horses were only classified based on detectable radiographic lesions. Therefore, we can not exclude the possibility that some lesions may not be radiographically visible, and eventually bias the results regarding a possible relation between bone markers and OC status.

The trend for lower IGF-I values found in the positive OC group of horses in our study is in accordance with previous results reported in literature [31, 60]. In a cross-sectional study with a single point blood sampling, which included radiographic examinations as a part of the protocol for stallion admission to Belgian Warmblood Studbook, lower values of IGF-I were also observed in the two-year-old horses’ group, which showed higher radiographic severity scores in what concerns osteoarticular status [61]. Additionally, lower IGF-I plasma concentrations were observed in pathological horses when compared to healthy ones, in a sequential study aiming at studying juvenile digital degenerative osteoarthropathy in the Ardenner breed [62]. Once this growth factor is involved in the mechanisms of bone growth and cartilage maturation, we can only speculate that the tendency for lower values in OC positive horses may have impaired the natural regression process of some OC lesions. The observed decrease in IGF-I concentrations from 12 to 18 months of age was probably associated to the decline in pasture quality and availability during fall, in Mediterranean conditions [35].

The hypothesis that relative hyperinsulinaeamia may be a contributory factor to equine OC was suggested by Henson et al. [63] from *in vitro* studies with fetal and neonate equine chondrocytes. Altered levels of circulating insulin may, therefore, have a direct endocrine effect on cartilage maturation through an increase on chondrocyte survival [63, 64]. In the present study, concentrate feeds were introduced in foals’ diet during the weaning and post-weaning periods, which occurred from six months onwards. Considering the observed trend for differences between OC-group status we can only speculate that higher insulin concentrations during this period could have been associated to a higher carbohydrate intake from diet, which may have led to alterations in the process of cartilage maturation and endochondral ossification and/or impaired the regression of some OC lesions. In fact, our results are consistent with the transient increase in insulin concentrations observed in positive OC foals between seven and 10 months of age, after the introduction of concentrate feeds in foals’ diet [60].

As for the SOS measurements we could not find any reports relating plasma leptin concentrations and OC status in the horse. Nevertheless, the increased values observed in the last two age-classes are in agreement with the change pattern reported by our research group [65].

Very few studies have investigated horse PTH concentrations under field conditions [66] and none during such a long period in the growing foal. The values found in the present study were in the range of values obtained for healthy adult horses and young foals [67, 68]. However, our results were lower than those observed by Sloet van Oldruitenborgh-Oosterban et al. [31], which can be clearly explained by the different type of assay used in this last study. In addition to the intact PTH molecule, the commercial kit used by this group of researchers, also measured the C-terminal/mid molecule fragments. Despite the different unit scale, our results regarding the trend for higher PTH values in the OC positive group were in agreement with the findings of Sloet van Oldruitenborgh-Oosterban et al. [31], who observed higher PTH concentrations in OC positive foals at four months of age. The results of our study regarding total Ca plasma concentrations were not very clear in what concerns OC status. The small changes in total Ca concentrations between age-classes observed in the OC negative group seem to be followed by the expected physiologic changes of PTH until the 18 months of age. Despite Ca^2+^ concentrations were not precisely determined, the results from this age-class onwards could be partially explained by hysteresis of PTH secretion [69, 70], as total Ca concentrations reflect also its ionized active form in the blood [71, 72].

The P_i_ and Mg concentrations were not influenced by OC status and followed the age-change pattern previously described [65]. Very few studies have investigated basal P_i_ and Mg plasma concentrations in relation to OC, but the lack of differences in P_i_ and Mg blood concentrations between positive and negative OC horses was also reported elsewhere [31].

The regularity of exercise conditions and the type of pasture surface, which were previously considered as risk factors for a poor osteoarticular status [52] were not investigated in the present study. Regarding exercise, and because all the horses had been exposed to similar conditions during the first months of life, we can only speculate that the different size of the paddocks during the post-weaning period may have provided different free exercise regimen, which could have had an additional effect on the latter OC status.

## Conclusions

Data from this study was obtained in real field conditions. In this regard, the conclusions drawn from the present research may be also useful to other breeds, managed in similar production systems. The presence of radiographic findings compatible with OC lesions at the onset of training was associated with changes in BW and WH growth patterns. In addition, OC positive horses presented a higher maturing index than healthy ones concerning BW growth model. Bone quality, as assessed by QUS, bone markers, growth factors and other metabolic variables changed markedly with age, and some trends were identified as being potentially related to OC status. This study provides indirect evidences that monitoring the foals’ growth during the first year of life may be of assistance in managing the occurrence of OC. Further prospective studies with a higher number of animals and controlled feed intake are warranted in order to strengthen the present findings.

## Supporting information

S1 Table Chemical composition and nutritive value of pastures (on DM basis)^1^ sampled in the four stud-farms during the spring (March, April and May). ^1^Values are presented as means ± SD. ^2^Number of samples. ^3^DE (digestible energy) and NE (net energy) were estimated according to INRA system. ^4^DP (digestible protein) and MADC (horse digestible crude protein) were estimated according to INRA system.
(DOCX)

S2 Table Chemical composition and nutritive value of commercial compound feeds (on DM basis)^1^ sampled in the four stud-farms. ^1^Values are presented as means ± SD. ^2^Number of analyzed samples. ^3^DE (digestible energy) and NE (net energy) were estimated according to INRA system. ^4^ DP (digestible protein) and MADC (horse digestible crude protein) were estimated according to INRA system.
(DOCX)

S3 Table Parameter estimates of the individual growth models fitted to body weight-age data set of the Lusitano horses included in the study (n = 34). ^a^ BW–body weight. ^b^ *A*–asymptotic value for BW as age approaches infinity (interpreted as mean BW at maturity); *b*–scaling parameter that defines the degree of maturity when age = 0 d (intercept on y axis); *k*–maturing index (rate that establishes the spread of the curve along time axis); *M*–determines the point of inflexion of the curve (for 0 < *M* < 1. M is undefined). ^c^ R^2^ correspond to a pseudo R^2^. calculated as 1 –(SS(Residual) / SS(Total corrected)). ^d^ RSD–residual standard deviation. ^e^ SE–approximate standard error.
(DOCX)

S4 Table Parameter estimates of the individual growth models fitted to withers height-age data set of the Lusitano horses included in the study (n = 34). ^a^ WH–withers height. ^b^ *A*–asymptotic value for WH as age approaches infinity (interpreted as mean WH at maturity); *b*–scaling parameter that defines the degree of maturity when age = 0 d (intercept on y axis); *k*–maturing index (rate that establishes the spread of the curve along time axis); *M*–determines the point of inflexion of the curve (for 0 < *M* < 1. M is undefined). ^c^ R^2^ correspond to a pseudo R^2^. calculated as 1 –(SS(Residual) / SS(Total corrected)). ^d^ RSD–residual standard deviation. ^e^ SE–approximate standard error.
(DOCX)

S5 Table Spearman’s correlation between body weight, withers height and girth measures of the Lusitano horses included in the study (n = 34). (DOCX)

S1 Fig Lateromedial views of the fetlocks taken at the end of the study. Example of the radiographic findings and their gradation (adapted from Dik et al. scoring system [10]). (a) Smooth flattening of the dorsoproximal aspect of the sagittal ridge of the third metacarpal bone—Graded 1; (b) Small indentation in the dorsoproximal aspect of the sagittal ridge of the third metacarpal bone—Graded 2; (c) Small fragment at the most dorsoproximal aspect of the sagittal ridge of the third metacarpal bone—Graded 3.
(TIF)

## References

[1] Martin-Rosset W. Growth and development in the equine In: Juliand V, Martin-Rosset W, editors. The growing horse: nutrition and prevention of growth disorders. EAAP series 114. Wageningen Academic Publishers, Wageningen, the Netherlands; 2005 pp.15–50.

[2] Valette J-P, Robert C, Denoix J-M. Use of linear and non-linear functions to describe the growth of young sport- and race-horses born in Normandie. Animal. 2008; 2: 560–5. 10.1017/S1751731107001462 22443570

[3] Kocher A, Staniar WB. The pattern of thoroughbred growth is affected by foal’s birth date. Livest Sci. 2013; 154: 204–14.

[4] Fradinho MJ, Bessa RJB, Ferreira-Dias G, Caldeira RM. Growth and development of the Lusitano horse managed on grazing systems. Livest Sci. 2016; 186: 22–8.

[5] Thompson KN, Baker JP, Jackson SG. The influence of supplemental feed on growth and bone development of nursing foals. J. Anim. Sci. 1988; 66: 1692–6. 10.2527/jas1988.6671692x 3403399

[6] Cymbaluk NF, Christison GI, Leach DH. Longitudinal growth analysis of horses following limited and ad libitum feeding. Equine Vet J. 1990; 22: 198–204. 2361509 10.1111/j.2042-3306.1990.tb04247.x

[7] Sandgren B, Dalin G, Carlsten J, Lundeheim N. Osteochondrosis in the tarsocrural joint and osteochondral fragments in the fetlock joints in Standardbred trotters. II. Body measurements and clinical findings. Equine Vet J. 1993; Suppl: 48–53.

[8] van Weeren PR, Sloet van Oldruitenborgh-Oosterbaan M, Barneveld A. The influence of birth weight, rate of weight gain and final achieved height and sex on the development of osteochondrotic lesions in a population of genetically predisposed Warmblood foals. Equine Vet J. 1999; Suppl: 26–30. 10.1111/j.2042-3306.1999.tb05310.x 10999657

[9] van Weeren PR, Jeffcott LB. Problems and pointers in osteochondrosis: twenty years on. Vet J. 2013; 197: 96–102. 10.1016/j.tvjl.2013.03.048 23639371

[10] Dik KJ, Enzerink E, van Weeren PR. Radiographic development of osteochondral abnormalities in the hock and stifle of Dutch Warmblood foals, from age 1 to 11 months. Equine Vet J. 1999; 31: 9–15. 10.1111/j.2042-3306.1999.tb05308.x 10999655

[11] Denoix J-M, Jeffcott LB, McIlwraith CW, van Weeren PR. A review of terminology for equine juvenile osteochondral conditions (JOCC) based on anatomical and functional considerations. Vet J. 2013; 197: 29–35. 10.1016/j.tvjl.2013.03.038 23683533

[12] Barneveld A, van Weeren PR. Conclusions regarding the influence of exercise on the development of the equine musculoskeletal system with special reference to osteochondrosis. Equine Vet J. 1999; Suppl. 31: 112–9. 10.1111/j.2042-3306.1999.tb05323.x 10999670

[13] Donabédian M, Fleurance G, Perona G, Robert C, Lepage O, Trillaud-Geyl C et al Effect of fast vs. moderate growth rate related to nutrient intake on developmental orthopaedic disease in the horse. Animal Res. 2006; 55: 471–86.

[14] Ytrehus B, Carlson CS, Ekman S. Etiology and pathogenesis of osteochondrosis. Vet Pathol. 2007;44: 429–48. 10.1354/vp.44-4-429 17606505

[15] Distl O. The genetics of equine osteochondrosis. Vet J. 2013;197: 13–8. 10.1016/j.tvjl.2013.03.036 23809989

[16] Lepage O, Carstanjen B, Uebelhart D. Non-invasive assessment of equine bone: an update. Vet J. 2001;161: 10–23. 10.1053/tvjl.2000.0541 11145827

[17] Carstanjen B, Lepage O, Detilleux J, Duboeuf F, Amory H. Use of multisite quantitative ultrasonography for non-invasive assessment of bone in horses. Am J Vet Res. 2002;63: 1464–9. 12371776 10.2460/ajvr.2002.63.1464

[18] Carstanjen B, Lepage O, Hars O, Langlois P, Duboeuf F, Amory H. Speed of sound measurements of the third metacarpal bone in young exercising Thoroughbred racehorses. Bone. 2003;33: 411–8. 13678783 10.1016/s8756-3282(03)00113-3

[19] Fradinho MJ, Vale AC, Bernardes N, Caldeira RM, Vaz MF, Ferreira-Dias G. Biomechanical properties of the equine third metacarpal bone: *in vivo* quantitative ultrasonography *vs ex vivo* compression and bending techniques. J Equine Vet Sci. 2015;35: 198–205.

[20] Price J, Jackson B, Gray J, Harris P, Wright I, Pfeiffer D et al Biochemical markers of bone metabolism in growing thoroughbreds: a longitudinal study. Res Vet Sci. 2001;71: 1–9. 10.1053/rvsc.2001.0496 11666146

[21] Reller E, Kivipelto J, Ott E. Age-related changes for serum bone metabolism markers in Thoroughbred and Quarter Horse foals. J Equine Vet Sci. 2003;23: 117–20.

[22] Billinghurst RC, Brama PAJ, van Weeren PR, Knowlton MS, McIlwraith CW. Evaluation of serum concentrations of biomarkers of skeletal metabolism and results of radiography as indicators of severity of osteochondrosis in foals. Am J Vet Res. 2004;65: 143–50. 14974569 10.2460/ajvr.2004.65.143

[23] Donabédian M, van Weeren PR, Perona G, Fleurance G, Robert C, Léger S et al Early changes in biomarkers of skeletal metabolism and their association to the occurrence of osteochondrosis (OC) in the horse. Equine Vet J. 2008;40: 253–9. 10.2746/042516408X273657 18267892

[24] Trumble TN, Brown MP, Merritt KA, Billinghurst RC. Joint dependent concentrations of bone alkaline phosphatase in serum and synovial fluids of horses with osteochondral injury: an analytical and clinical validation. Osteoarthr Cartilage. 2008;16: 779–86. 10.1016/j.joca.2007.11.008 18162418

[25] Ducy P. The role of osteocalcin in the endocrine cross-talk between bone remodelling and energy metabolism. Diabetologia. 2011;54: 1291–7. 10.1007/s00125-011-2155-z 21503740

[26] Ferron M, Lacombe J. Regulation of energy metabolism by the skeleton: osteocalcin and beyond. Arch Biochem Biophys. 2014;561: 137–46. 10.1016/j.abb.2014.05.022 24893146

[27] van der Eerden BCJ, Karperien M, Wit JM. Systemic and local regulation of the growth plate. Endocr Rev. 2003;24: 782–801. 10.1210/er.2002-0033 14671005

[28] Fortier LA, Kornatowski MA, Mohammed HO, Jordan MT, O'Cain LC, Stevens WB. Age-related changes in serum insulin-like growth factor-I, insulin-like growth factor-I binding protein-3 and articular cartilage structure in Thoroughbred horses. Equine Vet J. 2005;37: 37–42. 15651732 10.2746/0425164054406838

[29] Mackie EJ, Tatarczuch L, Mirams M. The skeleton: a multi-functional complex organ. The growth plate chondrocyte and endochondral ossification. J Endocrinol. 2011;211: 109–21. 10.1530/JOE-11-0048 21642379

[30] Savage CJ, McCarthy RN, Jeffcott LB. Effects of dietary phosphorus and calcium on induction of dyschondroplasia in foals. Equine Vet J. 1993;Suppl 16: 80–3.

[31] Sloet van Oldruitenborgh-Oosterban M, Mol J, Barneveld A. Hormones, growth factors and other plasma variables in relation to osteochondrosis. Equine Vet. J. 1999;Suppl 31: 45–54. 10.1111/j.2042-3306.1999.tb05313.x 10999660

[32] World Breeding Federation for Sport Horses (WBFSH). Studbook Rankings 2018 [Internet]. [cited 20 May 2019]. Available from: http://www.wbfsh.org/GB/Rankings/Breeder%20and%20Studbook%20rankings/2018.aspx

[33] Peel MC, Finlayson BL, McMahon TA. Updated world map of the Köppen-Geiger climate classification. Hydrol Earth Syst Sci. 2007;11: 1633–1644.

[34] Instituto Português do Mar e da Atmosfera, IP. Climate Normals 1981–2010 [Internet]. [cited 22 January 2019]. Available from: http://www.ipma.pt/pt/oclima/normais.clima

[35] Paço TA, Fradinho MJ. The role of extensive grazing systems in Southern Europe horse prodution In: Prochazka NT. Editor. Pastures: Dynamics, Economics and Management. Nova Science Publishers New York, NY; 2011 pp. 131–150.

[36] Hoyt S, Siciliano P. A comparison of ELISA and RIA techniques for the detection of serum osteocalcin in horses. Proceedings 16th Equine Nutr. Phys. Symp. 1999, pp. 351–352.

[37] Jackson BF, Goodship AE, Eastell R, Price JS. Evaluation of serum concentrations of biochemical markers of bone metabolism and insulin-like growth factor I associated with treadmill exercise in young horses. Am. J. Vet. Res. 2003;64: 1549–56. 14672435 10.2460/ajvr.2003.64.1549

[38] Cosden RS. Insulin-like growth factor-I in growing horses and RNA isolation from small articular cartilage samples. M.Sc. Thesis. Virginia Polytechnic Institute and State University. 2007. Available from: https://vtechworks.lib.vt.edu/handle/10919/34700

[39] McManus CJ, Fitzgerald BP. Effects of a single day of feed restriction on changes in serum leptin, gonadotropins, prolactin, and metabolites in aged and young mares. Domest Anim Endocrinol. 2000;19: 1–13. 10962194 10.1016/s0739-7240(00)00061-8

[40] Cartmill JA, Thompson DL, Gentry LR, Pruett HE, Johnson CA. Effects of dexamethasone, glucose infusion, adrenocorticotropin, and propylthiouracil on plasma leptin concentrations in horses. Domest Anim Endocrinol. 2003;24: 1–14. 12450621 10.1016/s0739-7240(02)00183-2

[41] Tinworth KD, Wynn PC, Boston RC, Harris PA, Sillence MN, Thevis M et al Evaluation of commercial available assays for the measurement of equine insulin. Domest Anim Endocrinol. 2011;41: 81–90. 10.1016/j.domaniend.2011.05.001 21741576

[42] Brown JE, Fitzhugh A Jr, Cartwright TC. A comparison of nonlinear models for describing weight-age relationship in cattle. J. Anim. Sci. 1976;42: 810–8.

[43] Perotto D, Cue RI, Lee AJ. Comparison of nonlinear functions for describing the growth curve of three genotypes of dairy cattle. Can J Anim Sci. 1992;72: 773–82.

[44] Richards FJ. A flexible growth function for empirical use. J Exp Bot. 1959;10: 290–300.

[45] Staniar WB, Kronfeld DS, Hoffman RM, Wilson JA, Harris PA. Weight prediction from linear measures of growing Thoroughbreds. Equine Vet J. 2004;36: 149–54. 15038438 10.2746/0425164044868585

[46] Jacquet S, Robert C, Valette J-P, Denoix J-M. Evolution of radiological findings detected in the limbs of 321 young horses, between the age of 6 and 18 months. Vet J. 2013;197: 58–64. 10.1016/j.tvjl.2013.03.042 23660154

[47] Baccarin RY, Pereira MA, Roncati NV, Bergamaschi RR, Hagen SC. Development of osteochondrosis in Lusitano foals: a radiographic study. Can Vet J. 2012;53: 1079–84. 23543926 PMC3447310

[48] Bernardes NFG. Estudo da influência do exercício, da idade e da presença de lesões de osteocondrose nos níveis séricos de biomarcadores ósseos no cavalo Lusitano linha Alter Real. M.Sc. Thesis. Faculdade de Medicina Veterinária / Instituto Superior de Agronomia, Universidade Técnica de Lisboa. 2008. Available from: https://www.repository.utl.pt/handle/10400.5/119

[49] Teixeira JAA. Avaliação radiográfica de osteocondrose como contributo na selecção de reprodutores equinos da raça Puro Sangue Lusitano. M.Sc. Thesis. Faculdade de Medicina Veterinária, Universidade Técnica de Lisboa. 2009. Available from: https://www.repository.utl.pt/handle/10400.5/1573

[50] Vander Heyden L, Lejeune JP, Caudron I, Detilleux J, Sandersen C, Chavatte P et al Association of breeding conditions with prevalence of osteochondrosis in foals. Vet Rec. 2013;172: 68 10.1136/vr.101034 23118049

[51] Robert C, Valette J-P, Jacquet S, Lepeule J, Denoix J-M. Study design for the investigation of likely aetiological factors of juvenile osteochondral conditions (JOCC) in foals and yearlings. Vet J. 2013;197: 36–43. 10.1016/j.tvjl.2013.03.039 23642464

[52] Lepeule J, Bareille N, Robert C, Ezanno P, Valette J-P, Jacquet S et al Association of growth, feeding practices and exercise conditions with the prevalence of developmental orthopaedic disease in limbs of foals. Prev Vet Med. 2009;89: 167–77. 10.1016/j.prevetmed.2009.02.018 19329202

[53] Lepeule J, Seegers H, Rondeau V, Robert C, Denoix J-M, Bareille N. Association of growth, feeding practices and exercise conditions with the severity of the osteo-articular status of limbs in French foals. Vet J. 2013;197: 65–71. 10.1016/j.tvjl.2013.03.043 23664071

[54] Carroll CL, Huntington PJ. Body condition scoring and weight estimation of horses. Equine Vet J. 1988;20: 41–5. 3366105 10.1111/j.2042-3306.1988.tb01451.x

[55] Carstanjen B, Duboeuf F, Detilleux J, Lepage O. Equine third metacarpal bone assessment by quantitative ultrasound and dual energy X-Ray absorptiometry: an *ex vivo* study. J Vet Med A. 2003;50: 42–77. 10.1046/j.1439-0442.2003.00495.x 12650508

[56] Firth EC, van Weeren PR, Pfeiffer DU, Delahunt J, Barneveld A. Effect of age, exercise and growth rate on bone mineral density (BMD) in third carpal bone and distal radius of Dutch Warmblood foals with osteochondrosis. Equine Vet J. 1999;Suppl 31: 74–8. 10.1111/j.2042-3306.1999.tb05317.x 10999664

[57] Rho J-Y, Kuhn-Spearing L, Zioupos P. Mechanical properties and the hierarchical structure of bone. Med Eng Phys. 1998;20: 92–102. 9679227 10.1016/s1350-4533(98)00007-1

[58] Clarke B. Normal Bone Anatomy and Physiology. Clin J Am Soc Nephrol. 2008;3: S131–9. 10.2215/CJN.04151206 18988698 PMC3152283

[59] Vervuert I, Winkelsett S, Christmann L, Bruns E, Hoppen H-O, Distl O et al Evaluation of the influences of exercise, birth date, and osteochondrosis on plasma bone marker concentrations in Hannoverian Warmblood foals. Am J Vet Res. 2007;68; 1319–23. 10.2460/ajvr.68.12.1319 18052735

[60] Baccarin RYA, Pereira MA, Roncati NV, Furtado PV, Oliveira CA, Hagen SCF. Identificação dos níveis séricos do fator de crescimento tipo insulina 1 em potros com osteocondrose. Pesq Vet Bras. 2011;31: 677–82.

[61] Verwilghen D, Busoni V, Gangl M, Franck T, Lejeune J-Ph, Vanderheyden L et al Relationship between biochemical markers and radiographic scores in the evaluation of the osteoarticular status of Warmblood stallions. Res Vet Sci. 2009;87: 319–28. 10.1016/j.rvsc.2009.02.002 19298987

[62] Lejeune J-Ph, Franck T, Gangl M, Schneider N, Michaux C, Deby-Dupont G et al Plasma concentration of Insulin-like Growth Factor I (IGF-I) in growing Ardenner horses suffering from Juvenile Digital Degenerative Osteoarthropathy. Vet Res Commun. 2007;31: 185–95. 10.1007/s11259-006-3385-2 17216321

[63] Henson FMD, Davenport C, Butler L, Moran I, Shingleton WD, Jeffcott LB et al Effects of insulin and insulin-like growth factors I and II on the growth of equine fetal and neonatal chondrocytes. Equine Vet J. 1997;29: 441–7. 9413716 10.1111/j.2042-3306.1997.tb03156.x

[64] Jeffcott LB, Henson FMD. Studies on growth cartilage in the horse and their application to aetiopathogenesis of dyschondroplasia (osteochondrosis). Vet J. 1998;156: 177–92. 9883086 10.1016/s1090-0233(98)80121-4

[65] Fradinho MJ, Mateus L, Bessa RJB, Caldeira RM, Ferreira-Dias G. Age-related changes of bone ultrasound measurements and metabolic indicators in the young horse. Livest Sci. 2018;211: 104–10.

[66] Azarpeykan S, Dittmer KE, Gee EK, Marshall JC, Wallace J, Elder P, Acke E, Thompson KG. Influence of blanketing and season on vitamin D and parathyroid hormone, calcium, phosphorus, and magnesium concentrations in horses in New Zealand. Domest Anim Endocrinol. 2016;56: 75–84. 10.1016/j.domaniend.2016.03.003 27131337

[67] Estepa JC, Aguilera-Tejero E, Mayer-Valor R, Almaden Y, Felsenfeld AJ, Rodriguez M. Measurement of parathyroid hormone in horses. Equine Vet J. 1998;30: 476–81. 9844965 10.1111/j.2042-3306.1998.tb04522.x

[68] Estepa JC, Garfia B, Gao P, Cantor T, Rodriguez M, Aguilera-Tejero E. Validation and clinical utility of a novel immunoradiometric assay exclusively for biologically active whole parathyroid hormone in the horse. Equine Vet J. 2003;35: 291–5. 12755433 10.2746/042516403776148246

[69] Toribio RE, Kohn CW, Sams RA, Capen CC, Rosol TJ. Hysteresis and calcium set-point for the calcium parathyroid hormone relationship in healthy horses. Gen Comp Endocrinol. 2003;130: 279–88. 12606270 10.1016/s0016-6480(02)00621-4

[70] Felsenfeld AJ, Rodriguez M, Aguilera-Tejero E. Dynamics of parathyroid hormone secretion in health and secondary hyperparathyroidism. Clin J Am Soc Nephrol. 2007; 2: 1283–305. 10.2215/CJN.01520407 17942777

[71] Berlin D, Aroch I. Concentrations of ionized and total magnesium and calcium in healthy horses: Effects of age, pregnancy, lactation, pH and sample type. Vet J. Vet J. 2009; 181, 305–11. 10.1016/j.tvjl.2008.03.014 18467135

[72] Toribio RE. Disorders of calcium and phosphate metabolism in horses. Vet Clin Equine. 2011; 27, 129–47. 10.1016/j.cveq.2010.12.010 21392658

