# Supplementary material for: Growth patterns, metabolic indicators and osteoarticular status in the Lusitano horse: A longitudinal study
Source: PLoS One. 2019 Jul 17;14(7):e0219900. doi: 10.1371/journal.pone.0219900 (PMC6636759; doi:10.1371/journal.pone.0219900)
Supplement: S1 Table — 1Values are presented as means ± SD. 2Number of samples. 3DE (digestible energy) and NE (net energy) were estimated according to INRA system. 4DP (digestible protein) and MADC (horse digestible crude protein) were estimated according to INRA system. (DOCX) [file pone.0219900.s001.docx]

S1 - Chemical composition and nutritive value of pastures (on DM basis)^1^ sampled in the four stud-farms during the spring (March, April and May).

|  | Months | | |
| --- | --- | --- | --- |
|  | March  (n=7)^2^ | April  (n=5)^2^ | May  (n=7)^2^ |
| DM, % | 14.5±2.5 | 20.3±3.2 | 45.2±28.8 |
| CP, % | 21.2±3.5 | 19.0±4.5 | 12.8±5.1 |
| CF, % | 18.2±3.3 | 20.4±4.6 | 29.0±8.8 |
| NDF, % | 36.0±4.7 | 46.2±10.8 | 55.6±18.4 |
| ADF, % | 23.2±3.6 | 27.7±2.8 | 37.5±9.7 |
| ADL, % | 5.1±1.4 | 6.7±3.3 | 8.1±1.5 |
| Ash, % | 11.9±1.1 | 12.2±2.6 | 9.4±2.0 |
| P, % | 0.50±0.06 | 0.39±0.06 | 0.30±0.10 |
| Ca, % | 1.05±0.12 | 0.85±0.36 | 0.88±0.42 |
| Mg, % | 0.27±0.03 | 0.27±0.07 | 0.18±0.05 |
| Zn, mg/kg | 33.4±6.5 | 30.4±8.0 | 33.0±10.9 |
| Cu, mg/kg | 8.0±2.2 | 6.6±1.5 | 7.9±3.1 |
| DE^3^, MJ/kg | 11.8±0.6 | 10.5±1.1 | 8.6±2.3 |
| NE^3^, MJ/kg | 7.1±0.5 | 6.7±0.7 | 5.5±1.2 |
| DP^4^, g/kg | 155±30 | 137±39 | 83±44 |
| MADC^4^, g/kg | 140±27 | 123±35 | 75±40 |

^1^Values are presented as means ± SD. ^2^Number of samples. ^3^DE (digestible energy) and NE (net energy) were estimated according to INRA system. ^4^DP (digestible protein) and MADC (horse digestible crude protein) were estimated according to INRA system.
