# Supplementary material for: Growth patterns, metabolic indicators and osteoarticular status in the Lusitano horse: A longitudinal study
Source: PLoS One. 2019 Jul 17;14(7):e0219900. doi: 10.1371/journal.pone.0219900 (PMC6636759; doi:10.1371/journal.pone.0219900)
Supplement: S2 Table — 1Values are presented as means ± SD. 2Number of analyzed samples. 3DE (digestible energy) and NE (net energy) were estimated according to INRA system. 4 DP (digestible protein) and MADC (horse digestible crude protein) were estimated according to INRA system. (DOCX) [file pone.0219900.s002.docx]

S2 - Chemical composition and nutritive value of commercial compound feeds (on DM basis)^1^ sampled in the four stud-farms.

|  | Commercial compound feeds (n=21)  Mean ± SD |
| --- | --- |
| DM, % | 86.4±2.9 |
| CP, % | 18.0±2.4 |
| CF, % | 12.1±1.7 |
| Ash, % | 8.1±1.7 |
| P, % | 0.54±0.09 |
| Ca, % | 1.22±0.53 |
| Mg, % | 0.22±0.03 |
| Zn, mg/kg | 89.0±29.9 |
| Cu, mg/kg | 13.8±6.4 |
| DE^3^, MJ/kg | 13.8±0.5 |
| NE^3^, MJ/kg | 8.8±0.5 |
| DP^4^, g/kg | 161±22 |
| MADC^4^, g/kg | 129±21 |

^1^Values are presented as means ± SD. ^2^Number of analyzed samples. ^3^DE (digestible energy) and NE (net energy) were estimated according to INRA system. ^4^ DP (digestible protein) and MADC (horse digestible crude protein) were estimated according to INRA system.
