# Supplementary material for: Growth patterns, metabolic indicators and osteoarticular status in the Lusitano horse: A longitudinal study
Source: PLoS One. 2019 Jul 17;14(7):e0219900. doi: 10.1371/journal.pone.0219900 (PMC6636759; doi:10.1371/journal.pone.0219900)
Supplement: S3 Table — a BW–body weight. b A–asymptotic value for BW as age approaches infinity (interpreted as mean BW at maturity); b–scaling parameter that defines the degree of maturity when age = 0 d (intercept on y axis); k–maturing index (rate that establishes the spread of the curve along time axis); M–determines the point of inflexion of the curve (for 0 < M < 1. M is undefined). c R2 correspond to a pseudo R2. calculated as 1 –(SS(Residual) / SS(Total corrected)). d RSD–residual standard deviation. e SE–approximate standard error. (DOCX) [file pone.0219900.s003.docx]

S3 Table – Parameter estimates of the individual growth models fitted to body weight-age data set of the Lusitano foals included in the study (n=34).

| Measure ^a^ | Stud | Parameters ^b^ | | | | R^2 c^ | RSD ^d^ |
| --- | --- | --- | --- | --- | --- | --- | --- |
|  |  | *A* (± SE^e^) | *b* (± SE^e^) | *k* (± SE^e^) | *M* (± SE^e^) |  |  |
|  |  |  |  |  |  |  |  |
| BW  (kg) | A  (n=10) | 584.3 | 0.9866 | 0.00086 | 0.519 | 0.990 | 13.2 |
|  |  | 533.4 | 0.9484 | 0.00121 | 0.712 | 0.987 | 15.2 |
|  |  | 587.9 | 0.9829 | 0.00090 | 0.573 | 0.993 | 11.2 |
|  |  | 558.7 | 0.9970 | 0.00062 | 0.428 | 0.977 | 17.2 |
|  |  | 456.9 | 0.9965 | 0.00103 | 0.429 | 0.967 | 19.4 |
|  |  | 470.3 | 0.9926 | 0.00139 | 0.448 | 0.979 | 16.8 |
|  |  | 548.2 | 0.9879 | 0.00062 | 0.499 | 0.981 | 15.3 |
|  |  | 427.7 | 0.9669 | 0.00148 | 0.580 | 0.983 | 13.5 |
|  |  | 448.7 | 0.9944 | 0.00132 | 0.462 | 0.986 | 12.5 |
|  |  | 571.2 | 0.9990 | 0.00060 | 0.452 | 0.979 | 16.3 |
|  |  |  |  |  |  |  |  |
|  | B  (n=9) | 587.1 | 0.8836 | 0.00212 | 1.053 | 0.990 | 16.2 |
|  |  | 510.0 | 0.9176 | 0.00222 | 0.932 | 0.951 | 30.1 |
|  |  | 631.5 | 0.9676 | 0.00110 | 0.731 | 0.983 | 20.0 |
|  |  | 665.0 | 0.9658 | 0.00104 | 0.709 | 0.976 | 23.7 |
|  |  | 628.9 | 0.9700 | 0.00119 | 0.673 | 0.992 | 14.2 |
|  |  | 516.2 | 0.9063 | 0.00166 | 1.023 | 0.989 | 15.2 |
|  |  | 472.8 | 0.7580 | 0.00266 | 1.580 | 0.995 | 10.2 |
|  |  | 507.6 | 0.9708 | 0.00149 | 0.731 | 0.989 | 14.3 |
|  |  | 500.1 | 0.8672 | 0.00222 | 1.044 | 0.970 | 23.3 |
|  |  |  |  |  |  |  |  |
|  | C  (n=6) | 554.4 | 0.9823 | 0.00168 | 0.640 | 0.981 | 17.1 |
|  |  | 538.2 | 0.8896 | 0.00193 | 0.922 | 0.997 | 8.5 |
|  |  | 600.3 | 0.9812 | 0.00145 | 0.681 | 0.997 | 9.1 |
|  |  | 598.7 | 0.9555 | 0.00151 | 0.794 | 0.997 | 7.4 |
|  |  | 721.9 | 0.9896 | 0.00071 | 0.634 | 0.997 | 8.2 |
|  |  | 689.1 | 0.9688 | 0.00094 | 0.730 | 0.991 | 13.5 |
|  |  |  |  |  |  |  |  |
|  | D  (n=9) | 592.4 | 0.9958 | 0.00091 | 0.546 | 0.988 | 15.4 |
|  |  | 544.0 | 0.9940 | 0.00119 | 0.559 | 0.977 | 19.6 |
|  |  | 617.8 | 0.9573 | 0.00083 | 0.558 | 0.958 | 22.6 |
|  |  | 454.6 | 0.9957 | 0.00176 | 0.553 | 0.980 | 17.0 |
|  |  | 447.8 | 0.9998 | 0.00191 | 0.540 | 0.969 | 21.5 |
|  |  | 548.5 | 0.9988 | 0.00072 | 0.465 | 0.967 | 21.2 |
|  |  | 518.8 | 0.9986 | 0.00171 | 0.597 | 0.971 | 25.7 |
|  |  | 434.9 | 0.9952 | 0.00157 | 0.523 | 0.971 | 19.6 |
|  |  | 427.0 | 0.9982 | 0.00145 | 0.495 | 0.966 | 19.2 |
|  |  |  |  |  |  |  |  |
|  | **Mean** | **544.0 ± 74.5** | **0.9635±0.0675** | **0.00135±0.00069** | **0.671±0.236** | **0.981** | **16.6** |

^a^ BW – body weight. ^b^ *A* – asymptotic value for BW as age approaches infinity (interpreted as mean BW at maturity); *b* – scaling parameter that defines the degree of maturity when age = 0 d (intercept on y axis); *k* – maturing index (rate that establishes the spread of the curve along time axis); *M* – determines the point of inflexion of the curve (for 0 < *M* < 1. M is undefined). ^c^ R^2^ correspond to a pseudo R^2^. calculated as 1 – (SS(Residual) / SS(Total _corrected_)). ^d^ RSD – residual standard deviation. ^e^ SE – approximate standard error.
