# Supplementary material for: Growth patterns, metabolic indicators and osteoarticular status in the Lusitano horse: A longitudinal study
Source: PLoS One. 2019 Jul 17;14(7):e0219900. doi: 10.1371/journal.pone.0219900 (PMC6636759; doi:10.1371/journal.pone.0219900)
Supplement: S4 Table — a WH–withers height. b A–asymptotic value for WH as age approaches infinity (interpreted as mean WH at maturity); b–scaling parameter that defines the degree of maturity when age = 0 d (intercept on y axis); k–maturing index (rate that establishes the spread of the curve along time axis); M–determines the point of inflexion of the curve (for 0 < M < 1. M is undefined). c R2 correspond to a pseudo R2. calculated as 1 –(SS(Residual) / SS(Total corrected)). d RSD–residual standard deviation. e SE–approximate standard error. (DOCX) [file pone.0219900.s004.docx]

S4 Table – Parameter estimates of the individual growth models fitted to withers height-age data set of the Lusitano foals included in the study (n=34).

| Measure ^a^ | Stud | Parameters ^b^ | | | | R^2 c^ | RSD ^d^ |
| --- | --- | --- | --- | --- | --- | --- | --- |
|  |  | *A* (± SE^e^) | *b* (± SE^e^) | *k* (± SE^e^) | *M* (± SE^e^) |  |  |
|  |  |  |  |  |  |  |  |
| WH  (cm) | A  (n=10) | 163.4 | 0.9832 | 0.00098 | 0.128 | 0.995 | 1.2 |
|  |  | 162.4 | 0.9604 | 0.00130 | 0.146 | 0.995 | 1.1 |
|  |  | 159.2 | 0.9009 | 0.00206 | 0.205 | 0.997 | 0.9 |
|  |  | 158.6 | 0.8999 | 0.00214 | 0.198 | 0.993 | 1.4 |
|  |  | 157.0 | 0.9767 | 0.00180 | 0.132 | 0.995 | 1.0 |
|  |  | 157.6 | 0.9242 | 0.00236 | 0.165 | 0.988 | 1.8 |
|  |  | 163.9 | 0.9754 | 0.00078 | 0.126 | 0.997 | 1.0 |
|  |  | 160.4 | 0.9859 | 0.00079 | 0.114 | 0.985 | 1.9 |
|  |  | 157.9 | 0.9716 | 0.00130 | 0.132 | 0.995 | 1.1 |
|  |  | 173.1 | 0.9956 | 0.00041 | 0.107 | 0.988 | 1.7 |
|  |  |  |  |  |  |  |  |
|  | B  (n=9) | 160.3 | 0.9291 | 0.00168 | 0.180 | 0.992 | 1.5 |
|  |  | 162.7 | 0.9507 | 0.00161 | 0.170 | 0.989 | 1.6 |
|  |  | 161.3 | 0.8165 | 0.00264 | 0.281 | 0.991 | 1.7 |
|  |  | 156.3 | 0.9454 | 0.00190 | 0.159 | 0.995 | 1.2 |
|  |  | 166.8 | 0.9674 | 0.00123 | 0.133 | 0.994 | 1.3 |
|  |  | 155.6 | 0.9182 | 0.00175 | 0.190 | 0.993 | 1.5 |
|  |  | 159.2 | 0.9627 | 0.00145 | 0.168 | 0.995 | 1.3 |
|  |  | 155.5 | 0.9473 | 0.00191 | 0.184 | 0.997 | 1.0 |
|  |  | 155.8 | 0.7630 | 0.00345 | 0.295 | 0.993 | 1.3 |
|  |  |  |  |  |  |  |  |
|  | C  (n=6) | 161.7 | 0.9072 | 0.00213 | 0.196 | 0.996 | 0.9 |
|  |  | 167.6 | 0.9762 | 0.00126 | 0.150 | 0.997 | 0.9 |
|  |  | 167.9 | 0.9479 | 0.00190 | 0.192 | 0.998 | 1.0 |
|  |  | 162.7 | 0.9526 | 0.00146 | 0.178 | 0.994 | 1.2 |
|  |  | 158.0 | 0.9137 | 0.00194 | 0.215 | 0.997 | 0.9 |
|  |  | 161.3 | 0.9403 | 0.00159 | 0.183 | 0.996 | 0.9 |
|  |  |  |  |  |  |  |  |
|  | D  (n=9) | 162.1 | 0.9870 | 0.00139 | 0.133 | 0.995 | 1.2 |
|  |  | 163.1 | 0.9856 | 0.00157 | 0.140 | 0.993 | 1.5 |
|  |  | 156.0 | 0.9812 | 0.00215 | 0.138 | 0.997 | 0.9 |
|  |  | 155.1 | 0.9852 | 0.00166 | 0.126 | 0.995 | 1.1 |
|  |  | 154.8 | 0.9471 | 0.00208 | 0.162 | 0.994 | 1.2 |
|  |  | 159.0 | 0.9764 | 0.00148 | 0.138 | 0.997 | 0.9 |
|  |  | 162.9 | 0.9758 | 0.00154 | 0.150 | 0.995 | 1.3 |
|  |  | 159.2 | 0.9990 | 0.00088 | 0.101 | 0.995 | 1.0 |
|  |  | 152.1 | 0.9544 | 0.00181 | 0.146 | 0.994 | 1.2 |
|  |  |  |  |  |  |  |  |
|  | **Mean** | **160.3 ± 2.7** | **0.9472±0.0360** | **0.00166±0.0004** | **0.163±0.029** | **0.994** | **1.2** |

^a^ WH – withers height. ^b^ *A* – asymptotic value for WH as age approaches infinity (interpreted as mean WH at maturity); *b* – scaling parameter that defines the degree of maturity when age = 0 d (intercept on y axis); *k* – maturing index (rate that establishes the spread of the curve along time axis); *M* – determines the point of inflexion of the curve (for 0 < *M* < 1. M is undefined). ^c^ R^2^ correspond to a pseudo R^2^. calculated as 1 – (SS(Residual) / SS(Total _corrected_)). ^d^ RSD – residual standard deviation. ^e^ SE – approximate standard error.
