# Supplementary material for: Growth patterns, metabolic indicators and osteoarticular status in the Lusitano horse: A longitudinal study
Source: PLoS One. 2019 Jul 17;14(7):e0219900. doi: 10.1371/journal.pone.0219900 (PMC6636759; doi:10.1371/journal.pone.0219900)
Supplement: S5 Table — (DOCX) [file pone.0219900.s005.docx]

S5 Table – Spearman’s correlation between body weight, withers height and girth measures of the Lusitano foals included in the study (n=34).

| Variable | Body Weight | Withers height | Girth |
| --- | --- | --- | --- |
| Body Weight | 1.00000 |  |  |
| *P* |  |  |  |
| n | 689 |  |  |
| Withers height | **0.97812** | 1.00000 |  |
| *P* | <.0001 |  |  |
| n | 637 | 651 |  |
| Girth | **0.98865** | **0.97780** | 1.00000 |
| *P* | <.0001 | <.0001 |  |
| n | 679 | 651 | 695 |
