# Supplementary figures and images for: Growth patterns, metabolic indicators and osteoarticular status in the Lusitano horse: A longitudinal study
Source: PLoS One. 2019 Jul 17;14(7):e0219900. doi: 10.1371/journal.pone.0219900 (PMC6636759; doi:10.1371/journal.pone.0219900)

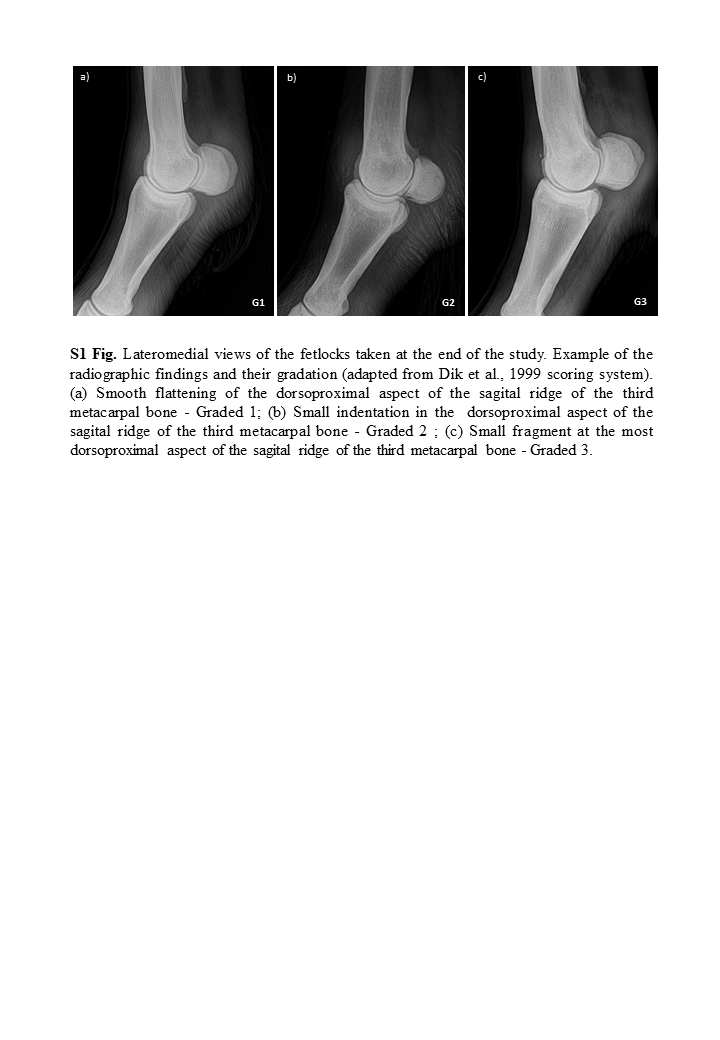

Supplement: S1 Fig — Example of the radiographic findings and their gradation (adapted from Dik et al. scoring system [10]). (a) Smooth flattening of the dorsoproximal aspect of the sagittal ridge of the third metacarpal bone—Graded 1; (b) Small indentation in the dorsoproximal aspect of the sagittal ridge of the third metacarpal bone—Graded 2; (c) Small fragment at the most dorsoproximal aspect of the sagittal ridge of the third metacarpal bone—Graded 3. (TIF) [file pone.0219900.s006.tif]
